# [Expression of Concern] Exogenous spermine attenuates diabetic kidney injury in rats by inhibiting AMPK/mTOR signaling pathway

**DOI:** 10.3892/ijmm.2026.5954

**Published:** 2026-08-12

**Authors:** Xinying Zhang, Li Zhang, Zhe Chen, Siwei Li, Bingbing Che, Ningning Wang, Junting Chen, Changqing Xu, Can Wei

Int J Mol Med 47: 27, 2021; DOI: 10.3892/ijmm.2021.4860

Following the publication of the above paper, it was drawn to the Editor's attention by an interested reader that the CD-2AP and podocin protein blots shown in Fig. 3A on p. 6 were strikingly similar, suggesting that the same data had apparently been used to represent the two different proteins.

The authors have been contacted by the Editorial Office to offer an explanation for the apparent duplication of data in this figure, and we are awaiting their response. Owing to the fact that the Editorial Office has been made aware of potential issues surrounding the scientific integrity of this paper, we are issuing an Expression of Concern to notify readers of this potential problem while the Editorial Office continues to investigate this matter further.

